# Comparative Three-Dimensional Analysis of Three Different Root Canal Obturation Techniques: An In Vitro Study

**DOI:** 10.7759/cureus.89885

**Published:** 2025-08-12

**Authors:** Khushboo Kumari, Amit Kumar, Rohit Miglani, Rajnish Kumar, Nazia Anwer, Manali Deb Barma, Prasanta Majumder

**Affiliations:** 1 Conservative Dentistry and Endodontics, Mithila Minority Dental College And Hospital, Darbhanga, IND; 2 Conservative Dentistry and Endodontics, Mithila Minority Dental College and Hospital, Darbhanga, IND; 3 Public Health Dentistry, Agartala Government Dental College and Indira Gandhi Memorial Hospital, Agartala, IND

**Keywords:** cone- beam computed tomography, root canal obturation, root canal therapy, single cone obturation, thermoplasticized gutta-percha, warm vertical compaction

## Abstract

Background

The outcome of a successful root canal treatment depends on effective debridement, shaping, and obturation of the root canal system. The process of obturation is a requisite for ensuring to provide a hermetic seal in order to prevent microbial re-infection. Among the various existing techniques, the single-cone technique, warm vertical compaction, and thermoplasticized gutta-percha are notably the most popular techniques used in endodontic procedures. While assessment of obturating techniques has hugely relied on 2D radio-imaging in the past, it has its limitations, such as distortion of image, superimposition. Therefore, this study was conducted to assess the efficacy of the three different obturating techniques using three 3D techniques of cone beam computed tomography (CBCT). The findings will aid clinicians for long-term, successful outcomes for root canal therapies.

Method

The current in vitro study evaluated the efficacy of three obturation techniques - single-cone, warm vertical compaction, and thermoplasticized gutta-percha - in 72 extracted human premolars with single canals. The teeth samples were disinfected, decoronated, and instrumented using Neo Endo rotary files. After random allocation (n=24 per group), canals were obturated using one of the three techniques. Specimens were scanned via CBCT and analyzed for adaptation, voids, and density in apical, middle, and coronal thirds by two blinded observers. Data were analyzed using one-way ANOVA and Bonferroni post hoc tests (α=0.05).

Results

The thermoplasticized technique showed significantly superior performance with 100%, 75%, and 75% void-free cases at apical, middle, and coronal levels, respectively (mean values: 0.10-0.20). Warm vertical compaction demonstrated moderate results (75% to 62.5% void-free), while the single-cone technique showed the highest void formation (50% to 37.5% void-free). Statistical analysis revealed highly significant differences between techniques (p=0.00).

Conclusion

The thermoplasticized gutta-percha technique produced the most consistent results with minimal voids, making it the most effective obturation method as compared to single-cone and warm vertical compaction.

## Introduction

Root canal therapy serves as a foundational procedure in contemporary dentistry, designed to preserve structurally compromised teeth by eliminating intraradicular infections and preventing their recurrence. The long-term success of endodontic treatment is critically dependent on meticulous canal preparation, effective microbial debridement, and the quality and completeness of obturation. The process of obturation plays an important role in providing a hermetic seal so as to prevent recurrent infections and leakage of restorative materials [1,2].

A variety of obturation techniques are currently employed in endodontic practice, including the single-cone and multiple-cone techniques as well as methods involving cold lateral condensation and warm compaction. Among these, cold lateral condensation remains the most commonly used approach due to its relative simplicity and cost-effectiveness. However, its limitations include the potential for void formation and inadequate adaptation to complex root canal anatomies [3]. The single-cone technique, a variation of cold lateral obturation, involves the use of a single gutta-percha cone matched to the final taper of the prepared canal, eliminating the need for accessory cones. This method offers advantages in terms of procedural simplicity and reduced chairside time, making it an attractive option in clinical settings [4]. In addition, warm vertical compaction is a widely adopted technique in endodontics, wherein gutta-percha is heated to a softened state to facilitate better flow and adaptation to the canal anatomy. However, despite its effectiveness, this method is time-consuming, and it also carries a risk of sealer extrusion, which may contribute to post-operative discomfort [5]. Among the more recent advancements, thermoplasticized gutta-percha techniques have demonstrated superior performance, with several studies reporting significantly fewer voids compared to both the single-cone and warm vertical compaction techniques [6].

Post-operative evaluation of root canal treatment is a critical step in assessing the immediate quality as well as integrity of the treatment, and also helps in determining the long-term outcome of the involved teeth. Radiographic assessment, in particular, has been an indispensable tool in endodontics, allowing clinicians to identify potential deficiencies such as voids, overfilling, or underfilling [7]. Traditionally, the evaluation of the quality of endodontic treatment has relied heavily on 2D periapical radiographs. While providing valuable measurements and information, there are certain limitations to it, such as distortion of image, superimposition of anatomical structures, and inability to accurately assess the 3D morphology of the root canals [8]. The introduction of cone beam computed tomography (CBCT) for dentistry in the late 1990s [9] led to a breakthrough in dental imaging. CBCT offers a non-invasive, 3-D visualization of teeth and their surrounding structures with high spatial resolution. It provides detailed cross-sectional views, resulting in a more precise assessment of root canal morphology and the quality of obturation within the root canal system [10]. Therefore, this in vitro study aims to comparatively analyze the quality of root canal treatments achieved by three different obturation techniques: single-cone technique, warm vertical compaction, and thermoplasticized GP obturation technique. The current study seeks to provide valuable insights into the efficacy of each technique in achieving a homogeneous root canal filling, ultimately contributing to evidence-based practices.

## Materials and methods

Study design

The current study is an in vitro study conducted in the Department of Conservative Dentistry and Endodontics at Mithila Minority Dental College and Hospitals with prior institutional ethical committee approval (EC/NEW/INST/2023/4152/Ref 17). The study adhered to the Declaration of Helsinki principles and followed the Checklist for Reporting In Vitro Studies (CRIS) guidelines [11].

Sample size calculation

The sample size was calculated a priori with G*Power (Ver. 3.1, Heinrich-Heine-Universität Düsseldorf, Düsseldorf, Germany) with an effect size of 0.3 from a reference study [12], significance level (α) of 0.05, and 80% power; the required sample size was 72 (24 per group).

Inclusion and exclusion criteria

The current study included extracted human premolar teeth, either right or left, that possessed a single canal. Only teeth with fully formed apices and no visible signs of fracture or craze lines were considered eligible. Additionally, the root canals of the selected teeth were required to exhibit minimal to no curvature. Teeth were excluded from the study if they were grossly decayed and deemed unsuitable for instrumentation. Specimens showing severe root curvature or dilaceration were also omitted. Furthermore, any evidence of cervical or apical root resorption led to exclusion. Teeth that were found to be fractured or cracked during visual inspection were likewise excluded from the study.

Methodology

A total of 72 extracted human premolar teeth with single canals, fully formed apices, and minimal to no curvature of roots were collected. The samples were thoroughly rinsed under running tap water to remove residual blood and saliva. Initially, they were disinfected by immersing in 5% sodium hypochlorite (Parcan, Septodont Healthcare India Pvt Ltd., Mumbai, India) for 24 hours and then stored in normal saline to maintain hydration and avoid desiccation. To standardize the root lengths, all teeth were decoronated at the cementoenamel junction using a diamond disc under water cooling. The root length was standardized to approximately 16 mm. The working length was determined by inserting a #10 K-file (Dentsply Maillefer, Ballaigues, Switzerland) into the canal until the tip was visible at the apex and subtracting 0.5 mm. The canals were initially prepared using a #15 K-file (Dentsply Maillefer) to establish apical patency. This was followed by rotary instrumentation using the Neo Endo system, employing a crown-down technique. Irrigation was performed with 2 mL of 3% sodium hypochlorite (Parcan, Septodont Healthcare India Pvt Ltd.) between each file. Following preparation, the canals were dried using absorbent paper points.

The 72 specimens were randomly divided into three groups of 24 teeth each, based on the obturation technique to be employed: Group 1 (single-cone technique); Group 2 (warm vertical compaction using Orikam Fast Pack Pro-Down Pack Device (Orikam Healthcare, Gurgaon, India)); Group 3 (Thermoplasticized Gutta-Percha using Denjoy I-Fill GP Obturation Unit (Denjoy Dental Co. Ltd., Changsha, China).

**Figure 1:**
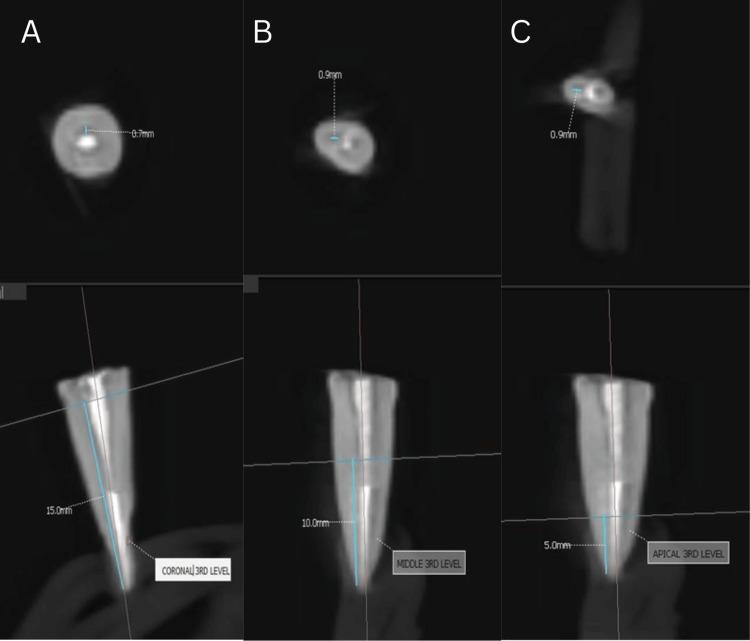
CBCT analysis of single cone technique. CBCT images showing obturation with the single-cone technique in the (A) coronal third, (B) middle third, and (C) apical third.

**Figure 2:**
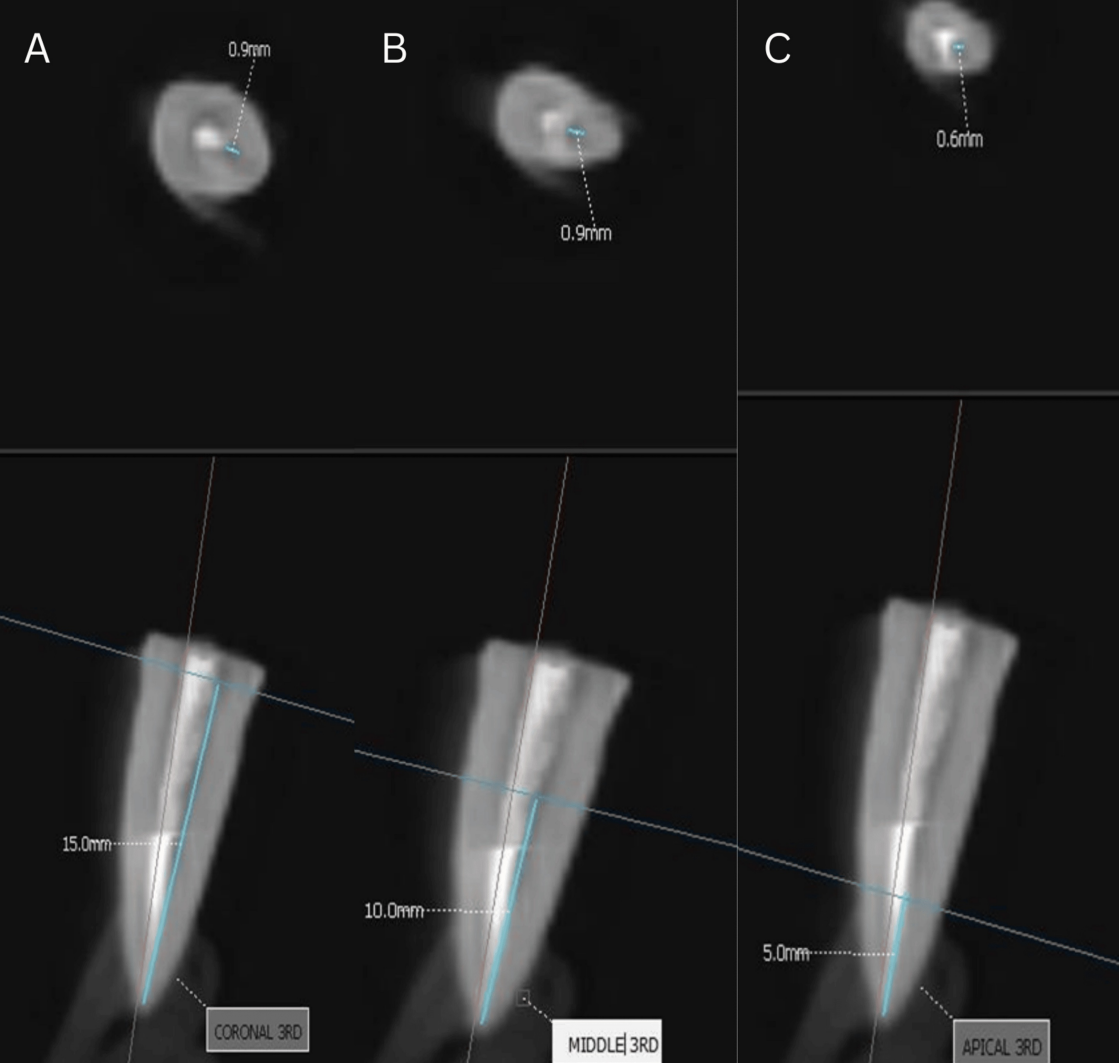
CBCT analysis of warm vertical compaction technique CBCT images showing obturation with the warm vertical compaction technique in the (A) coronal third, (B) middle third, and (C) apical third.

**Figure 3:**
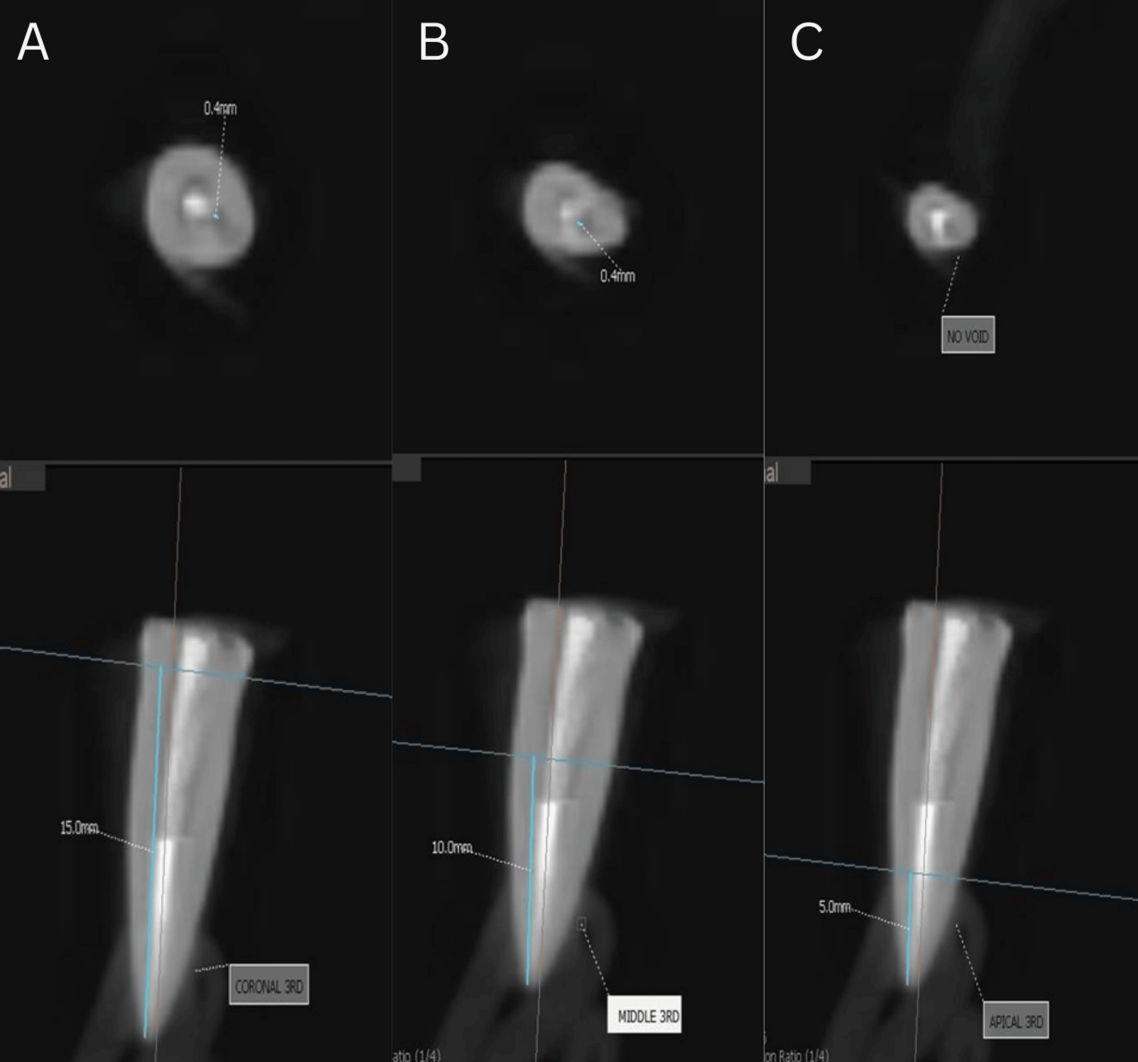
CBCT analysis of thermoplasticized gutta-percha technique CBCT images showing obturation with thermoplasticized gutta-percha technique in the (A) coronal third, (B) middle third, and (C) apical third.

Obturation was performed in Group 1 (Figure 1) using a single gutta-percha cone corresponding to the size of the master apical file, with sealer (Dentsply AH Plus Root Canal Sealing Material) applied to the canal walls. For Group 2 (Figure 2), gutta-percha was compacted vertically using the Orikam down-pack device. The procedure involved sectional heating and condensation of gutta-percha to achieve a dense fill. Thermoplasticized gutta-percha was injected into the canal using a thermoplastic delivery system to ensure uniform fill in Group 3 (Figure 3).

After obturation, specimens were embedded in wax slabs to stabilize them during imaging. Each slab contained teeth, arranged in a row, to facilitate uniform scanning. All specimens were subjected to CBCT imaging using standardized parameters. The images were analyzed in three horizontal sections: apical third, where evaluation focused on the adaptation and homogeneity of the obturation material near the apex; middle third, where assessment included the detection of voids and the uniformity of obturation; and for coronal third, the analysis targeted the density of the obturation and any presence of air gaps or voids. To ensure accuracy and inter-observer reliability, the CBCT images were independently assessed by two of the authors, both of whom were calibrated prior to evaluation. Inter-observer agreement was high (Cohen’s κ = 0.91), indicating substantial reliability. To determine the percentage of obturated volume, the calculations for the coronal third, middle third, and apical third were conducted independently. The inner area of the void space was measured and multiplied by the thickness of the slice to obtain the volume of the void. The percentage volume (POV) was calculated with the formula ($a-b &times; 100/a$), where $a$ is the root canal space volume and $b$ is the void space volume.

Statistical analysis

The data was compiled and analyzed using IBM SPSS Version 23.0 (Armonk, NY: IBM Corp). Normality of the data was assessed using the Shapiro-Wilk test, and all variables were found to follow a normal distribution (p > 0.05). Therefore, parametric tests were applied for further comparisons. A chi-square test was done to compare the presence of voids. One-way ANOVA test was conducted to compare the three obturation techniques, followed by a Bonferroni post hoc test. A significance level of p <0.05 was set.

## Results

Table 1 shows the comparison between the technique used and the presence of voids at the apical, middle, and coronal levels. At the apical third, the thermoplasticized technique demonstrated superior performance, with 100% of samples (n = 8) exhibiting no voids, indicating a highly effective seal in this critical region. Both the single-cone and warm vertical compaction techniques showed 75% (n = 6) of samples with no voids. Small voids (<0.5 mm) were detected in 25% (n = 2) of the single-cone technique group and in 12.5% (n = 1) of the warm vertical compaction group. A larger void (>0.5 mm and <1 mm) was present in only 12.5% (n = 1) of the warm vertical compaction samples. The difference among the techniques was statistically significant (chi-square = 36.96, p = 0.00), suggesting the thermoplasticized technique provided a significantly better apical seal. At the middle third of the canal, the thermoplasticized technique again demonstrated the best performance, with 75% (n = 6) of samples showing no voids. This was followed by warm vertical compaction, where 62.5% (n = 5) of the samples exhibited void-free obturation, and single-cone, which showed the lowest void-free rate at 50% (n = 4). Small voids were identified in 25% (n = 2) of samples in the thermoplasticized, single-cone, and warm vertical compaction groups. Larger voids (>0.5 mm - <1 mm) were present in 25% (n = 2) of the single-cone group, 12.5% (n = 1) of samples in the warm vertical compaction group, but were absent in the thermoplasticized group. The differences among the groups were statistically significant (chi-square = 62.66, p = 0.00). 

**Table 1:** Comparison between the technique used and the presence of voids at apical, middle and coronal level. The chi-square test was used to compare the techniques used and the presence of voids at the apical, middle, and coronal levels. *p-value <0.05 is considered statistically significant.

Level	Technique	No voids	Small voids (<0.5 mm)	Voids (>0.5 mm - < 1 mm)	Chi-square value	p-value
Apical level	Single-cone technique (n = 8)	75% (n = 6)	25% (n = 2)	0% (n = 0)	36.96	0.00*
	Warm vertical compaction (n = 8)	75% (n = 6)	12.5% (n = 1)	12.5% (n = 1)		
	Thermoplasticized technique (n = 8)	100% (n = 8)	0% ( n = 0)	0% (n = 0)		
Middle level	Single-cone technique (n = 8)	50% (n = 4)	25% (n = 2)	25% ( n = 2)	62.66	0.00*
	Warm vertical compaction (n = 8)	62.5% (n = 5)	25% (n = 2)	12.5% (n = 1)		
	Thermoplasticized technique (n = 8)	75% (n = 6)	25% ( n = 2)	0% (n = 0)		
Coronal level	Single-cone technique (n = 8)	37.5% (n = 3)	50% (n = 4)	12.5% (n = 1)	74.71	0.00*
	Warm vertical compaction (n = 8)	37.5% (n = 3)	37.5% (n = 3)	25% (n = 2)		
	Thermoplasticized technique (n = 8)	75% (n = 6)	12.5% (n = 1)	12.5% (n = 1)		

At the coronal third, the thermoplasticized technique maintained its superior performance, with 75% (n = 6) of samples demonstrating no voids. In contrast, both the single-cone and warm vertical compaction groups had only 37.5% (n = 3) of samples void-free. Small voids were most frequent in the single-cone group (50%, n = 4), followed by warm vertical compaction (37.5%, n = 3), and thermoplasticized group (12.5%, n = 1). Large voids were observed among 12.1% (n = 1) of samples in the single-cone technique, 25% (n = 2) of samples in warm vertical compaction, and 12.5% (n = 1) in the thermoplasticized technique. The observed differences at the coronal level were also found to be statistically significant (chi-square = 74.71, p = 0.00), with the thermoplasticized group showing significantly fewer voids overall.

Table 2 compares the effectiveness of three techniques based on their mean values, standard deviation, and statistical significance (p-value) at the apical, middle, and coronal levels. The findings indicate that the thermoplasticized technique consistently produces the least void formation, with mean values of 0.10 at the apical, 0.20 at the middle, and 0.10 at the coronal level. In contrast, the single-cone technique results in the highest void formation, with mean values of 0.39 at apical, 0.72 at middle, and 0.73 at coronal, particularly showing increased voids in the middle and coronal regions. The warm vertical technique falls in between, with mean values of 0.31, 0.44, and 0.49 at the apical, middle, and coronal levels, respectively. The standard deviation values indicate that the thermoplasticized technique provides more consistent results, as it has lower variability compared to the other two methods. The p-value (0.00) is highly significant (HS) across all comparisons, confirming that the observed differences between the techniques are statistically meaningful. Overall, the thermoplasticized technique is the most effective in minimizing void formation, making it the preferred choice, while the single technique is the least effective, and the warm vertical technique offers moderate performance.

**Table 2:** Mean comparison between the techniques used. One-way ANOVA was done to assess the mean between the three groups. *p-value <0.05 is considered statistical significant

Groups	N	Apical third (mean±SD)	Middle third (mean±SD)	Coronal third (mean±SD)
Group 1	24	0.39±0.7	0.72±0.13	0.73±0.15
Group 2	24	0.31±0.15	0.44±0.08	0.49±0.16
Group 3	24	0.10±0.12	0.20±0.13	0.10±0.81
p-value		0.00*	0.00*	0.00*

Table 3 presents the post hoc comparison of obturation techniques across the apical, middle, and coronal thirds of the root canal. In the apical section, a statistically significant difference was observed between the single-cone and thermoplasticised groups (mean difference = 0.29, p = 0.001) and between warm vertical and thermoplasticised groups (0.21, p = 0.001), while the difference between single-cone and warm vertical techniques was not statistically significant (p = 0.063). In both the middle and coronal sections, all intergroup comparisons showed statistically significant differences (p = 0.001), with the highest mean difference noted between the single-cone and thermoplasticised groups in the coronal section (0.63, p = 0.001). Overall, the thermoplasticised technique demonstrated significantly higher values across all root sections compared to the other techniques.

**Table 3:** Post hoc comparison of obturation techniques across apical third, middle third, and coronal third. Bonferroni post hoc test was applied. A p-value of <0.05 was considered statistically significant.

Section	Group Comparison	Mean Difference	p-value
Apical	Single − Warm vertical	0.08	0.063
	Single − Thermoplasticised	0.29	0.001
	Warm vertical − Thermoplasticised	0.21	0.001
Middle	Single − Warm vertical	0.28	0.001
	Single − Thermoplasticised	0.52	0.001
	Warm Vertical − Thermoplasticised	0.24	0.001
Coronal	Single − Warm vertical	0.24	0.001
	Single − Thermoplasticised	0.63	0.001
	Warm Vertical − Thermoplasticised	0.39	0.001

## Discussion

The precision of root canal obturation is a critical determinant factor for endodontic treatment outcomes, as it ensures effective sealing of the root canal and prevents reinfection. The current study compared the efficacy of three different obturation techniques in minimizing void formations at apical, middle, and coronal levels, a key element influencing the sealing ability of obturation materials. Among the three techniques, the thermoplasticized technique demonstrated superior performance with the least void formations across the apical, middle, and coronal thirds. This technique achieved a void-free fill in 100% (n=8) of the cases at the apical third, 75% (n =6) at the middle third, and similarly 75% (n=6) at the coronal third. These findings were found to be consistent with previous studies in producing dense fills with minimal voids [13-16].

Apart from CBCT, other evaluating methods, such as dye, have also shown consistent findings where the thermoplasticized obturation technique exhibited better sealing capacity [17]. This could be attributed to the ability of the thermoplasticized technique to soften and allow the flow of gutta-percha into simple as well as complex canal irregularities, likely contributing to its superior adaptation and homogeneity. Apart from these, a previous study shows that factors such as maximum inert core material and phase transformations are also reasons for the superiority of the thermoplasticized technique [18].

The warm vertical compaction technique showed moderate performance, with a noticeable reduction in void-free obturations from the apical third at 75% (n=6) to the coronal third at 37.5% (n=3). This could be due to difficulties in heat transfer and condensation efficiency in the coronal portion. Though the technique allows for controlled compaction and has demonstrated improved sealing ability over single-cone obturations in prior studies, operator dependency and technique sensitivity may affect consistency, especially in upper canal segments [19].

The single-cone technique resulted in the highest void formation, particularly in the middle and coronal thirds. This may be due to the limited ability of a single gutta-percha cone to adapt closely to canal walls, especially in oval or irregular canals. Although this technique is simple, quick, and often preferred in clinical practice, its limitations in achieving a void-free fill highlight the importance of proper case selection and potential adjunctive use of bio-ceramic sealers to improve adaptation [20]. Previously generated evidence suggests that obturation techniques that use pressure syringes or injectable devices for obturations also provide better endodontic outcomes [21].

The thermoplasticized technique consistently exhibited the lowest mean void volumes across all three levels in the current study. These minimal void values indicate that thermoplasticized obturation achieves a dense and well-adapted fill throughout the entire canal length. The ability of thermoplasticized gutta-percha to flow into canal irregularities when heated and compacted ensures a more complete fill, which is especially critical in areas with anatomical complexities.

CBCT imaging proved to be a valuable tool in this study, allowing three-dimensional assessment of obturation quality with high resolution and reproducibility. The current study produces evidence-based, plausible results to document the effective obturation technique in endodontic treatment. Beyond localized effects, chronic oral infections have been increasingly linked to systemic diseases, including cardiovascular disorders, diabetes mellitus, adverse pregnancy outcomes, and even autoimmune conditions [22]. Therefore, ensuring a dense, void-free obturation is not merely a technical goal but a clinical necessity with broader health implications.

Clinical implications

The findings of this study have direct clinical relevance in guiding the selection of obturation techniques for improved endodontic outcomes. The significantly superior performance of the thermoplasticized technique in minimizing void formations, especially at the critical apical third, reinforces its utility in cases requiring optimal sealing to prevent reinfection and ensure long-term success. Its ability to flow into canal irregularities allows for more complete canal obturation, making it particularly suitable for anatomically complex or curved canals.

Limitations

Since the study is in vitro, it may not replicate the complexities of in vivo conditions such as variable canal anatomy, the presence of moisture, and patient-related factors. The sample size used in the current study was also limited, which might affect the generalizability of the results. Additionally, the study did not assess the sealing ability or long-term performance of the obturation techniques under thermocycling or bacterial leakage models.

## Conclusions

Within the limits of the study, it is revealed that among the three, the thermoplasticized technique demonstrated superior performance, showing the least void formation and the most consistent adaptation of the obturating material across all levels. Warm vertical compaction showed moderate results, with a progressive increase in voids toward the coronal third, indicating technique sensitivity and operator dependency. The single cone technique exhibited the highest incidence of voids, especially in the middle and coronal regions, underscoring its limitations in achieving a dense three-dimensional fill, particularly in canals with complex morphology. While this study offers valuable insights, future research incorporating curved canals, different sealer types, and dynamic conditions would provide a more comprehensive understanding. Clinicians are encouraged to adopt advanced obturation methods for better clinical outcomes.
